# Equity-centered dementia prevention: lessons from a community-embedded intergenerational model

**DOI:** 10.3389/frdem.2026.1823303

**Published:** 2026-09-17

**Authors:** Phaedra Bell, Bruce Miller, Brian Lawlor

**Affiliations:** 1 Department of Neurology, University of California, San Francisco, San Francisco, CA, United States; 2 Edward and Pearl Fein Memory and Aging Center Founding Director, Global Brain Health Institute, UCSF, San Francisco, San Francisco, CA, United States; 3 Global Brain Health Institute, Trinity College Dublin, Dublin, Ireland

**Keywords:** community partnership, dementia prevention, health equity, implementation science, intergenerational programs, linguistic inclusion, social connection

## Abstract

Dementia prevention requires policies that address upstream, modifiable risk factors—particularly loneliness, depressed mood, and low social participation—which disproportionately affect linguistically diverse and historically marginalized communities. This Perspective argues that linguistic inclusion, cultural responsiveness, and trust-building practices should be treated as core quality domains in dementia prevention policy and delivery. We describe the Multimodal Intergenerational Social Contact Intervention (MISCI), a community-embedded program designed to reduce loneliness and depressed mood—established risk factors for dementia—through culturally meaningful intergenerational engagement. While MISCI outcomes are limited to feasibility, acceptability, and descriptive trends, we integrate these findings with existing literature to derive policy and systems recommendations. These include reimbursement pathways for social connection interventions, strengthened language access standards, equity-stratified reporting, and cross-sector partnerships. Aligning policy mechanisms with community-embedded programs may support scalable, culturally responsive approaches to reducing dementia risk while advancing health equity.

## Introduction

Dementia burden is rising globally, yet a meaningful proportion of risk is attributable to modifiable factors, including social isolation, loneliness, and depression (Livingston et al., 2020; U.S. Surgeon General, 2023; National Academies of Sciences, Engineering, and Medicine, 2020; World Health Organization, n.d.). These risk factors are unevenly distributed, disproportionately affecting linguistically diverse and historically marginalized populations due to structural barriers in access, communication, and trust (National Institutes of Health, 2017; U.S. Surgeon General, 2023; National Academies of Sciences, Engineering, and Medicine, 2020).

Emerging evidence suggests that interventions targeting social connection and mood may contribute to dementia risk reduction pathways, even when not explicitly designed as dementia-specific interventions (Livingston et al., 2020; Hickin et al., 2021; Burnes et al., 2019). This Perspective situates intergenerational social connection programs within a broader dementia prevention framework by linking psychosocial risk reduction with equity-centered implementation strategies, including constructs of social inclusion and intergroup connection (Pittinsky et al., 2011). To our knowledge, this is among the first perspectives to link intergenerational social connection interventions with equity-centered dementia prevention policy.

### Empirical anchor: MISCI

The Multimodal Intergenerational Social Contact Intervention (MISCI) pairs older adults experiencing loneliness with students to co-create culturally meaningful projects. MISCI is not a dementia-specific clinical intervention; rather, it is designed to address psychosocial risk factors—loneliness and depressed mood—that are associated with increased dementia risk (Livingston et al., 2020; U.S. Surgeon General, 2023; National Academies of Sciences, Engineering, and Medicine, 2020).

Mixed-methods program evaluation demonstrated feasibility, high acceptability, and descriptive trends toward improved loneliness, mood, and sense of purpose. These outcomes were measured using validated tools (e.g., UCLA Loneliness Scale, PHQ-2, Ryff Psychological Well-being Scales) (Hughes et al., 2004; Kroenke et al., 2003; Ryff and Keyes, 1995). Spanish-language triads increased engagement among participants with limited English proficiency, underscoring the importance of linguistic concordance in improving participation and experience (Diamond et al., 2019).

Importantly, MISCI outcomes are preliminary and descriptive. The program evaluation was not designed to test causal effects on dementia risk, but rather to inform implementation feasibility and identify mechanisms—such as social connection, cultural relevance, and linguistic concordance—that may contribute to downstream risk reduction. Therefore, recommendations presented below draw on both MISCI insights and the broader evidence base.

### Conceptual framework

The Equity-Centered Dementia Prevention Framework (Figure 1) was developed to guide interpretation of MISCI findings and integration with implementation science and health equity literature. The framework is adapted from established models including RE-AIM and CFIR, with an explicit equity lens (Glasgow et al., 2019; Damschroder et al., 2022; Shelton et al., 2020).

**Figure 1:**
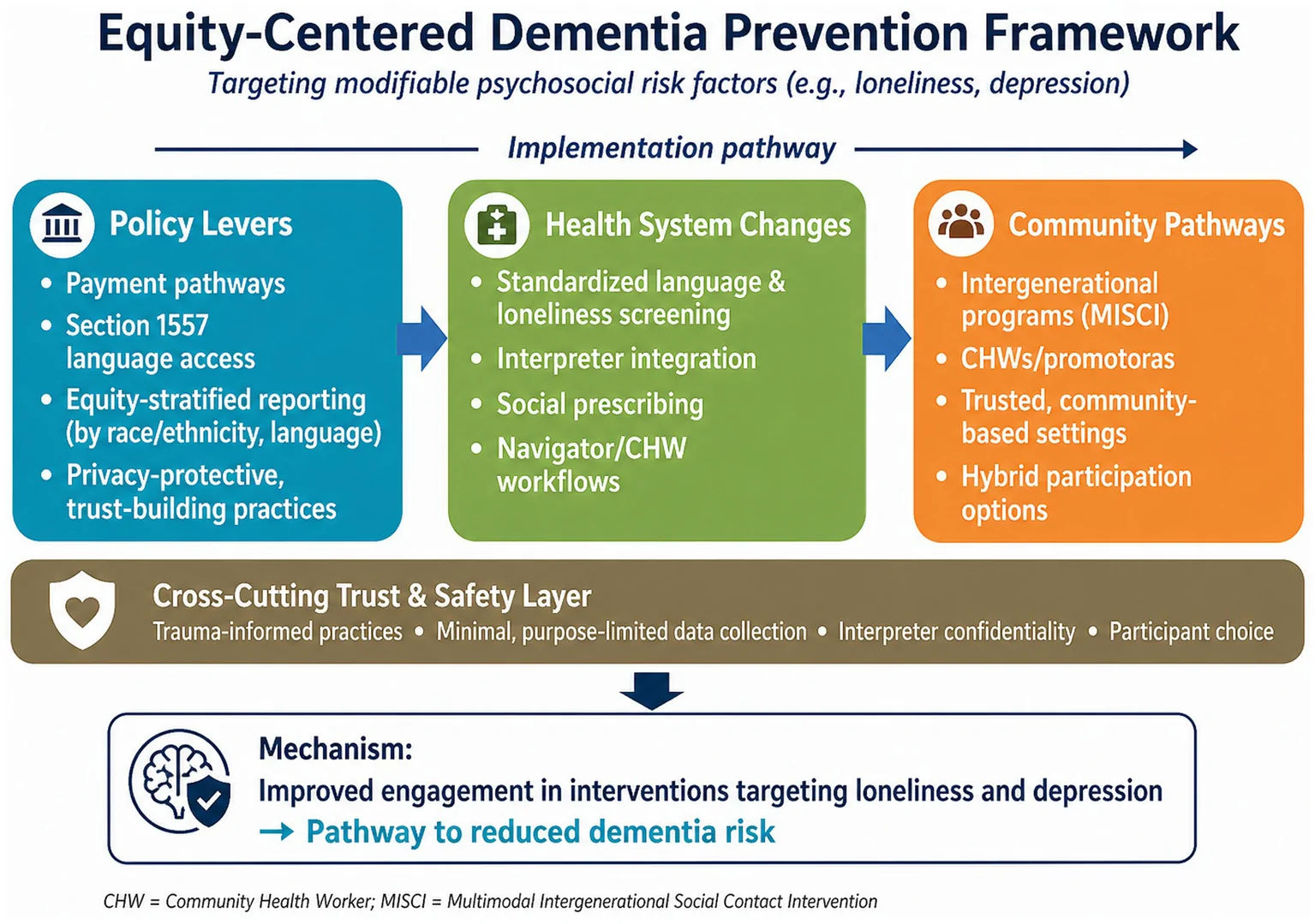
Equity-centered dementia prevention framework. The framework illustrates how policy levers, health system changes, and community pathways interact to improve engagement in interventions targeting modifiable psychosocial risk factors, thereby contributing to dementia risk reduction.

It links three domains:

- Policy levers (e.g., reimbursement, language access enforcement)
- Health system workflows (e.g., screening, referral pathways)
- Community pathways (e.g., intergenerational programs)

A cross-cutting layer emphasizes trust, trauma-informed practices, and privacy protections. In this context, “equity-stratified” refers to stratification of outcomes by sociodemographic variables (e.g., race/ethnicity, preferred language), enabling identification of disparities that may reflect inequities.

### Policy recommendations

Because MISCI identifies implementation features that improve engagement with interventions targeting loneliness and depression—established dementia risk factors—policy recommendations focus on scaling these features through system and policy mechanisms. Policy recommendations are derived from both MISCI implementation insights and existing literature (Table 1).

**Table 1:** Equity-centered dementia prevention actions and metrics.

Level	Priority actions	Equity metrics
Policy	Activate reimbursement for community-based prevention	Equity-stratified participation rates
Health system	Standardize loneliness and language screening	Screening and referral completion rates
Community	Deploy bilingual navigators/CHWs	Enrollment, engagement, and retention among participants with limited English proficiency (LEP), stratified by preferred language

Metrics should be interpreted as process and outcome measures, with equity assessed through stratified analyses across sociodemographic groups.

First, reimbursement pathways for social connection and navigation services are critical, as financial sustainability is a major barrier to scaling interventions targeting loneliness and mood (Centers for Medicare and Medicaid Services, 2023; Centers for Medicare and Medicaid Services, 2025). Second, strengthening language access enforcement under Section 1557 is essential to ensure equitable participation and quality of care for individuals with limited English proficiency (U.S. Department of Health and Human Services, Office for Civil Rights, 2024). Third, equity-stratified reporting should be implemented to monitor disparities in access and outcomes.

Privacy-protective and trauma-informed practices are foundational for engagement, particularly among populations that may experience distrust due to structural inequities or policy environments (Bernstein et al., 2021; Artiga et al., 2019; Page and Flores, 2020; Substance Abuse and Mental Health Services Administration, 2014). These practices support dementia prevention indirectly by enabling sustained participation in interventions that target modifiable risk factors.

Finally, formalizing cross-sector partnerships between health systems and community-based organizations is necessary to deliver culturally responsive programs at scale, as health systems alone often lack the infrastructure and trust relationships required for effective outreach.

### Systems recommendations

Health system recommendations focus on operationalizing dementia risk reduction through routine care processes.

Systems should standardize screening for loneliness and preferred language, integrate interpreter services, and establish referral pathways to community-based programs such as MISCI (National Academies of Sciences, Engineering, and Medicine, 2020; Diamond et al., 2019). Bilingual navigators and community health workers can support engagement, particularly for populations with limited English proficiency.

Monitoring should include RE-AIM-informed metrics stratified by race/ethnicity and language to assess reach, effectiveness, and equity (Glasgow et al., 2019; Shelton et al., 2020). These process and outcome measures (Table 1) should be interpreted alongside disparity analyses to determine whether equitable access and benefit are achieved.

### Implementation roadmap

The proposed 12–18-month implementation timeline reflects an aspirational pathway for well-resourced systems. However, timelines will vary substantially depending on infrastructure, partnerships, and policy environment.

Health systems may begin with readiness assessments and pilot programs, while broader policy changes—such as reimbursement reform and enforcement of language access standards—require coordination with governmental entities. A phased, context-sensitive approach is recommended, with early emphasis on pilot testing and partnership development, followed by scale-up informed by evaluation data.

### Limitations

MISCI findings are based on descriptive program evaluation without a control group, limiting causal inference regarding effects on dementia risk. Sample size and geographic concentration may limit generalizability.

Recommendations presented here integrate MISCI insights with broader literature; however, the translation from psychosocial outcomes to dementia prevention remains indirect and requires further empirical validation. Additional limitations include potential variability in implementation across settings, resource constraints in rural or under-resourced systems, and challenges in sustaining cross-sector partnerships.

Future research should include controlled trials, longitudinal follow-up, objective social network measures, and economic evaluations to assess scalability and return on investment.

## Conclusion

MISCI illustrates how community-embedded, culturally responsive programs can address modifiable psychosocial risk factors associated with dementia. While evidence remains preliminary, integrating such programs with policy and health system infrastructure offers a promising pathway for equitable dementia prevention.

Linguistic inclusion, cultural responsiveness, and trust-building are not ancillary features but core components of effective prevention strategies. Aligning policy levers, health system workflows, and community partnerships can support scalable approaches that reduce dementia risk while advancing equity. These findings highlight the importance of aligning implementation strategies with policy mechanisms to translate psychosocial risk reduction into population-level dementia prevention.

## Data Availability

The data supporting the conclusions of this article will be made available by the author upon reasonable request.
